# Impact of genetic relatedness on reproductive behavior in *Pelvicachromis pulcher*, a biparental cichlid fish with mutual mate choice and ornamentation

**DOI:** 10.1007/s00114-023-01842-z

**Published:** 2023-05-04

**Authors:** Leonie Gussone, Anna Hüllen, Simon Vitt, Ulrike Scherer, Timo Thünken

**Affiliations:** 1 Institute of Evolutionary Biology and Ecology, University of Bonn, An der Immenburg 1, 53121 Bonn, Germany; 2 Department of Anthropology, University of California, Santa Barbara, CA USA; 3 Institute of Zoology, University of Hamburg, Martin-Luther-King Platz 3, 20146 Hamburg, Germany; 4 Department of Biology and Ecology of Fishes, Leibniz Institute of Freshwater Ecology and Inland Fisheries, Müggelseedamm 310, 12587 Berlin, Germany; 5 Faculty of Life Sciences, Humboldt-Universität zu Berlin, Invalidenstrasse 42, 10115 Berlin, Germany

**Keywords:** Inbreeding preference, Kin selection, Inclusive fitness benefits, Male mate choice, Sexual selection, Female ornamentation, Cichlid fishes

## Abstract

Inbreeding can result in inbreeding depression. Therefore, many species seek to avoid inbreeding. However, theory predicts that inbreeding can be beneficial. Accordingly, some species tolerate inbreeding or even prefer mating with close relatives. Evidence for active inbreeding, i.e., kin-mating preference was reported in the biparental African cichlid fish *Pelvicachromis taeniatus.* Related mating partners revealed better parental cooperation due to kin selection, a potential benefit of inbreeding. In this study, we investigated kin-mating preference in a genetically diverse, outbred F2-lab population of *Pelvicachromis pulcher*, a closely related species to *P. taeniatus*. Like *P. taeniatus*, this species shows mutual ornamentation and mate choice as well as intense biparental brood care. The F1 *P. pulcher* generation had revealed signs of inbreeding depression but no inbreeding avoidance. We studied mating behavior and aggression in trios consisting of a male *P. pulcher*, an unfamiliar sister, and an unfamiliar, unrelated female. Because the study focused on kin-mating patterns, female pairs were matched for body size and coloration. The results provide no evidence for inbreeding avoidance but rather suggest inbreeding preference. We also found no significant impact of inbreeding on offspring survival. The results suggest no inbreeding avoidance in *P. pulcher*; however, the strength of inbreeding preference and inbreeding depression seems to be variable. We discuss possible causes for this variation like context-dependent inbreeding depression. The number of eggs positively correlated with female body size and coloration. Furthermore, the female aggressiveness was positively correlated with female coloration indicating that coloration signal female dominance and quality.

**Supplementary Information:**

The online version contains supplementary material available at 10.1007/s00114-023-01842-z.

## Introduction

Sexual selection is an evolutionary process that imposes strong selection pressure on behavioral and morphological traits (Andersson & Simmons, 2006). Darwin (1871) defined sexual selection as the advantage of some individuals over other same-sex individuals in a reproductive context. It can be distinguished between intersexual selection, which includes, e.g., mate choice for attractive ornaments, or intra-sexual selection which includes, e.g., contest competition for weaponry and symbols of status (Kokko et al., 2006). Therefore, sexual selection often results in the evolution of visual, olfactory, or acoustic ornaments to directly link the owners’ genetic quality or condition with its appearance (Darwin, 1871). Research on intersexual selection has mainly focused on the choice of males by females, but male mate choice recently received more attention (Herdman et al., 2004; Scherer & Schuett, 2018; Schlupp, 2021). Since females in many species invest relatively more in reproduction (Trivers, 1972) and have lower potential reproductive rates (Clutton-Brock & Vincent, 1991), females are expected to be the choosier sex. While females invest high energy to produce few oocytes, males provide the less costly spermatocytes in higher quantity (Bateman, 1948). However, if males invest in parental care or females vary in quality, males should also show mating preferences (Parker, 1983; Sargent et al., 1986). For example, in biparental species, both sexes are involved in intensive brood and offspring care (Thünken et al., 2010). Therefore, males are expected to be choosy as well due to increased reproduction costs (Kokko & Johnstone, 2002).

Mating between closely related individuals is known as inbreeding (Ballou, 1983). Because inbreeding can increase homozygosity of recessive deleterious alleles in the offspring (Hanna Kokko & Ots, 2006; Pusey & Wolf, 1996), the potential fitness costs can be very high, i.e., inbreeding depression (Charlesworth & Charlesworth, 1987). For instance, inbred individuals show reduced fecundity (Radwan, 2003), decreased survival (Fessehaye et al., 2007), or reduced reproductive success (Willoughby et al., 2019). Although inbreeding depression seems to be expressed most strongly in early life stages (Pusey & Wolf, 1996), it may also show consequences for adults such as sperm deformities (Brown et al., 1993), lower fertility (Su et al., 1996), or decreased courtship frequency (Waldmann & McKinnon, 1993). Although inbreeding depression can have profound negative effects and results often in inbreeding avoidance, some species have been shown to tolerate inbreeding or even prefer mating with close relatives (Daniel & Rodd, 2016; Langen et al., 2011; Nichols, 2017; Thünken et al., 2007). Active inbreeding avoidance is expected to evolve when the risk of inbreeding is high and when inbreeding is associated with inbreeding depression (Pike et al., 2021). Accordingly, inbreeding avoidance mechanisms evolve when the probability is high to encounter relatives during reproductive periods as present in species with small population sizes and low dispersal (Pusey & Wolf, 1996). However, the strength of inbreeding depression is not constant, but may vary; e.g., continuous inbreeding may lead to purging of deleterious alleles in inbred population, and then, inbreeding depression is negligible (Hedrick, 1994).

Mating with kin can be also beneficial because it can increase individual inclusive fitness (Kokko & Ots, 2006). Hamilton’s rule predicts better cooperation among relatives (Hamilton, 1964), which might be also true for costly parental cooperation. Accordingly, mating with kin can reduce the sexual conflict over care and improve parental cooperation and thus quality of care (Thünken et al., 2007). Furthermore, parental investment is typically predicted to increase with parent’s relatedness to dependent offspring. Therefore, optimal parental investment may be directly influenced by kinship between parents (Duthie et al., 2016; Gow et al., 2019). Because inbred offspring is more closely related to the parents than outbred offspring, this may increase parental investment of related parents. All in all, inbreeding avoidance, tolerance, or preference depends on the relative cost and benefits of inbreeding (Kokko & Ots, 2006).

The African cichlid fish *Pelvicachromis taeniatus*, which exhibits biparental brood care and is known for its monogamous behavior, has been observed to display a preference for mating with close kin. Despite originating from an isolated, inbred population (as demonstrated by Langen et al., 2011), there is no evidence of inbreeding depression among these fish, as shown by Thünken et al. in 2007. Inbreeding seems to be an advantageous strategy in *P. taeniatus* because related parents have been shown to be more cooperative and invested more in their offspring than unrelated parents (Thünken et al., 2007). This suggests that genetic relatedness plays an important role during mate choice. In this study, we investigated the kin-mating behavior in a genetically diverse lab population of *Pelvicachromis pulcher*, a closely related species to *P. taeniatus*. *P. pulcher* is a cave-breeding cichlid fish from West Africa with biparental brood care, mutual mate choice, and a monogamous mating system (Martin & Taborsky, 1997; Scherer, 2019). We found evidence for inbreeding depression with respect to juvenile survival and growth and female fecundity in F1 generation inbred fish (unpublished data). Here, we investigated the reproductive behavior in outbred fish of the F2 generation. We arranged trios consisting of a male *P. pulcher*, an unfamiliar sister, and an unfamiliar, unrelated female, and documented male-female courtship behavior as well as female-female and male-female aggression until final pair formation. Furthermore, we compared the survival rate of the resulting inbred and outbred offspring. When inbreeding preference appears to be a common trait in the *Pelvicachromis* group, we expect *P. pulcher* to show a similar inbreeding preference as *P. taeniatus*. However, considering to the previously shown negative effects of inbreeding in *P. pulcher*, we expect inbreeding avoidance to avoid these costs.

In *P. taeniatus*, female color intensity and size predict female fecundity, and both female traits play a role in male mate choice (Baldauf et al., 2009; Baldauf et al., 2010). To control for variation in these traits, the related and unrelated females were matched in body size and coloration. To examine whether female coloration signals female dominance and quality also in *P. pulcher*, we correlated egg number and female aggression with color intensity.

## Materials and methods

### Experimental animals

Our study species, *Pelvicachromis pulcher* (Boulenger, 1901), the rainbow krib, is a cichlid endemic to West Africa. This species inhabits rivers and streams and shows a conspicuous sexual dimorphism and dichromatism. Males and females differ in size, shape, and coloration. Males are larger than females and show a red to pinkish or yellow ventral and throat coloration. The smaller females are colored conspicuously cherry red to purple on the lateral side (Martin & Taborsky, 1997). *P. pulcher* is a predominantly socially monogamous cichlid fish with biparental brood care (Martin & Taborsky, 1997; Scherer, 2019). The breeding pairs form territories which they defend aggressively against intruders (Scherer, 2019). Eggs are spawned within breeding cavities where the embryos hatch after 2 to 3 days. After another 5 days, the so-called wrigglers (remain wriggling with their body on the ground) reach the free-swimming stage. The fry is guarded by both parental fish (Scherer, 2019).

We used the second generation of a genetically diverse lab population (unpublished data) as experimental animals. Fish were bred at the Institute of Evolutionary Biology and Ecology of the University of Bonn. All individuals were maintained in mixed-sex full-sibling groups in glass tanks (50 × 30 × 30 cm; length × width × height; l × w × h) equipped with sand, water plants (*Lemna minor* and *Ceratophyllum demersum*), and a filter (model: Gully filter; Hobby). The water temperature of the tanks was kept at 24 ± 1 °C and the light-dark cycle was set to 12:12 h. Adjacent tanks were separated by an opaque PVC plate to prevent visual contact. Prior to the experiment, fish were fed with two different feeding regimes. One group was assigned a homogenous feeding treatment and the second group a clumped feeding treatment (Schons et al., 2022). Both groups were fed with pellet food (0.25 pellets/fish; Rift Lake Red Cichlid Pellets S by Vitalis Aquatic Nutrition).

### Ethical note

The present study adheres to the ASAB/ABS guidelines for the use of animals in research, as well as to the legal requirements of Germany and was conducted in accordance with German laws for animal experiments. Experiments were approved by the regional office for nature, environment, and consumer protection North-Rhine Westphalia (LANUV NRW, reference no. 84e02.04.2015.A580 and 81-02.04.2021.A199).

### Experimental set-up

**Fig. 1:**
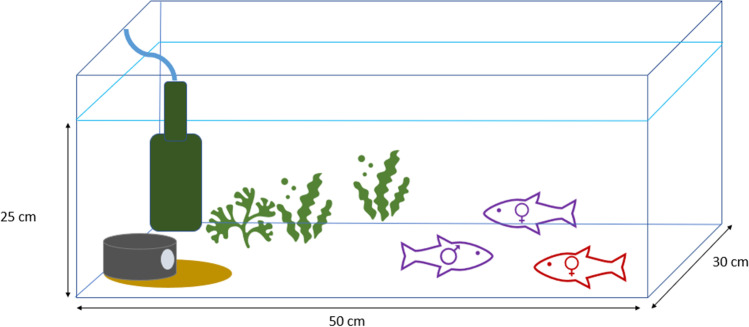
Experimental set-up for mate choice experiment with a trio. Related fish shown in purple and unrelated female shown in red. The breeding cavity is shown in the left corner, and the filter is shown in the left back corner

The male mating preference experiment was conducted in two identical experimental units. During the first experimental unit, 30 trials were conducted, and during the second experimental unit, 24 trials were conducted. The experiment was conducted by two observers, following an exact protocol to guarantee comparable data. All tanks (50 × 30 × 30 cm; l × w × h) were equipped with sand, water plants for shelter, filter (model: Gully filter; Hobby, Germany), and a ceramic cave as breeding cavity (Fig. 1). Experimental conditions were identical to holding conditions (12:12h light:dark, 24 ± 1 °C). Experimental fish were fed 6 days a week with a mixture of defrosted *Artemia*, Chironomidae, Culicidae, and Chaoboridae larvae.

Each male was presented with two unfamiliar females, a related and a non-related female. In both experimental units, a total of 46 males from 11 families and 108 females from 15 families were used. From those 46 males, 8 males were used twice but paired with different females.

### Measurement of body and color variables

Before the experiment, the standard lengths of all individuals were measured (tip of the mouth to the base of the caudal fin) to an accuracy of 1 mm. Therefore, the fish were carefully taken out of the water by hand and placed on graph paper. Afterwards, all individuals were placed in a small plastic box filled with water on a fine scale (Sartorius, ED 153-CW) to weigh each fish to the nearest milligram. Additionally, the body condition index (BCI) was calculated following Bolger and Connolly (1989) using the formula:

$$BCI=\frac{Weight\ast 100}{{Standard\ length}^3}$$

To quantify the LAB chromaticity, photographs of females were taken using a standardized set-up including a photo box before each experiment. The photo box, made of Plexiglas (9.5 × 15 × 7 cm, l × w × h), was placed on a table. Two light sources (6W LED lamps, Toshiba LDRC1665WE7EUD, 32°, 6500K) were used for standardized illumination, and photographs were taken using a Nikon D5000 camera with a macro objective (AF-S Micro Nikkor 105 mm 1:28 G). Each fish was placed carefully in the water-filled photo box and held in place using a plastic pipette. Photographs were saved in RAW format and color standards (Munsell color standard chip) as well as a size indicator was included on each photograph (for methodological details, see Vitt et al., 2022).

**Fig. 2:**
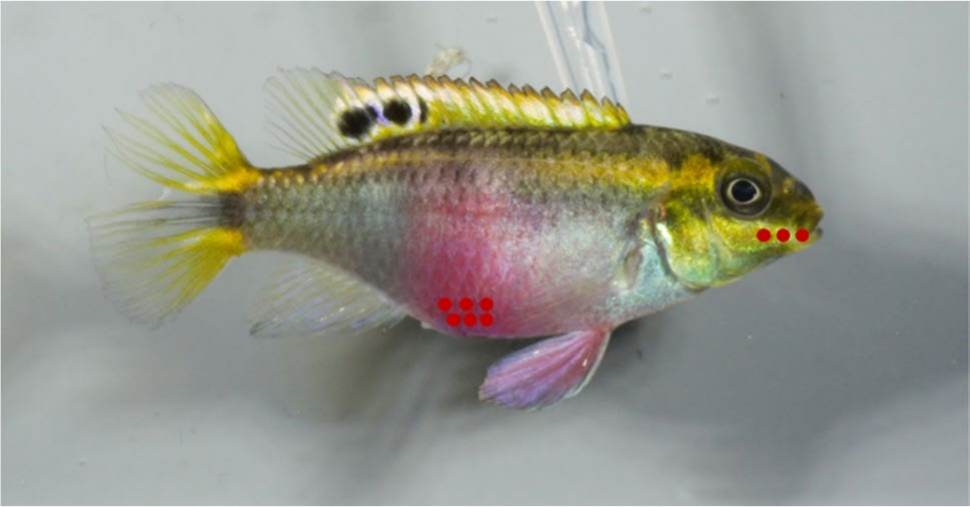
A female *P. pulcher*. Red dots show the points where LAB values were determined

To analyze the intensity of coloration (LC), the program Adobe Photoshop CS4 Extended (Version 11.0.2) was used. After the brightness of all pictures was standardized using the white standard on the photographs and light temperature was adjusted to the light temperature of the light sources (6500K), the coloration was measured in form of LAB values by applying the CIELab color space, which is commonly used to study fish coloration (Craig and Foote 2001; Meuthen et al. 2018). CIELab values separate brightness (L*) from color (a*, b*) (Chen & Hao, 2004). We measured six points from the abdomen and three points from the mouthpart, which were afterwards averaged for each body part (Fig. 2). The LAB chromaticity (LC) value was calculated by applying the formula according to Robertson (1977):

$$LAB\ chromaticity\ (LC)=\sqrt{a^2+b2}$$

Adult male *P. pulcher* were transferred to individual experimental tanks using a 1-L plastic box. Males were kept in the experimental tanks for 3 days without disturbance to give them the opportunity to establish territories including the prepared ceramic caves. On day 4, two adult females (i.e., one unfamiliar full-sibling female and one unfamiliar non-related female) were transferred to the experimental tanks. Because the study focused on the mating preferences with respect to genetic relatedness, female pairs were matched for body size and coloration. Related and unrelated females did not significantly differ in size (related female: 3.72 cm ± 0.30 cm; unrelated female: 3.70 cm ± 0.30 cm; paired *t*-test: *t* = − 0.59, *p* = 0.56) and color intensity on the abdomen (related female: 14.63 ± 7.21; unrelated female: 14.69 ± 5.63; paired *t*-test: *t* = 0.33, *p* = 0.74) or the mouthpart (related female: 57.33 ± 16.60; unrelated female: 57.00 ± 15.88; paired *t*-test: *t* = 1.15, *p* = 0.26). The two females were released simultaneously at the center of the tank. All three fish were kept without disturbance for 1 day. On the following day, the behavioral observations were started. Each tank was observed for 5 min per day in a random order. The observers (LG and AH) were blind with respect to kinship treatment. The females were distinguished by differences in the number of points on the dorsal fin. Courtship behaviors (count of body shaking) and aggression (attacks) were recorded for all three individuals. The mate choice experiment was considered as successful when a mating pair was formed. In 40 out of the 54 trials, the male showed a clear choice for one female, i.e., the chosen female entered the cave, and the not chosen female was hiding in the plants or behind the filter. To minimize stress on the breeding pair, the unselected female was promptly transferred back to the original tank on the same day. When no choice was made after 9 observational days (in 14 cases), the trial was terminated.

After a choice was made, the breeding cave was checked daily for egg clutches by carefully lifting the cave under water. Every egg clutch was photographed under water, and the number of eggs were counted. Additionally, the number of days until spawning were noted. The tanks were checked daily for free-swimming fry. Seven days after free-swimming fry was observed outside the breeding cavity, the number of individuals was counted. The fry was siphoned off the tank carefully into a bucket by using a plastic tube. After counting the individuals, the fry was transferred back into the breeding tank. The survival rate of each clutch was calculated by using the formula:

$$Survival\ rate\ \left(\%\right)=100\ast \left(\frac{Number\ of\ individuals}{Number\ of\ eggs}\right)$$

### Statistical analysis

Data were analyzed using R 4.0.3 (R Core Team, 2021). All models were fit as (G) LMM using the lme4-package (Bates et al., 2015). As random factors, the family of males and females, the ID of males, and the tank number were included in all LMMs and GLMMs to control for multiple uses of families and males. Since no difference in the influence of the two feeding regimes could be detected in preliminary analysis on the number of eggs (LM: *t* = 0.099, *p* = 0.922), survival rate of offspring (LM: *t* = 0.802, *p* = 0.428), female belly coloration (LM: *t* = 0.164, *p* = 0.87), and the female mouth coloration (LM: *t* = − 0.75, *p* = 0.455), it was not included as random factor. Non-significant variables were removed stepwise from the LMM in the order of their statistical relevance using the backward elimination procedure of the step function in the lmerTest package (Kuznetsova et al., 2017).

First, we investigated the effects of the relatedness on male mate choice (yes/no) using generalized mixed effect models (GLMM) with a binomial distribution. To analyze the effects of female body size and coloration on the mate choice decision, GLMMs were used. In this analysis, the difference between the chosen female and the not chosen female was used. Before investigating possible effects on the number of eggs and the survival rate of offspring, normal distribution of the residuals was tested using the performance package (Lüdecke et al., 2021). Then, we used LMMs to investigate the effects of relatedness to females on the days until a mating decision was made. We then used LMMs to explore the effects of the relatedness to the chosen females, the female body size, and the female coloration on the number of eggs as well as on the survival rate of offspring. Furthermore, we analyzed the effects of female relatedness, body size, and coloration on female-female aggression and the effects of the courtship behavior of females on the survival rate of offspring using LMMs. For the analysis of female-female aggression, all females showing no female-female aggression were excluded (*N* = 49/80). The coloration of each female showing aggression towards another female was used. The female aggression was calculated as the average attack from one female towards the other female per day per female. The female-female aggression differed significantly from a normal distribution and was successfully transformed with the Box-Cox power transformation. For the analysis of the survival rate, all data points with no courtship behavior or survival rate equals 0 were excluded from the analysis (*N* = 16/40). Finally, we analyzed the effects of the relatedness and the choice on male aggression (yes/no) towards females using GLMMs. Here, all males showing no aggression towards females were excluded (*N* = 48/80). The marginal *R*² was calculated using the performance package (Lüdecke et al., 2021).

## Results

**Fig. 3:**
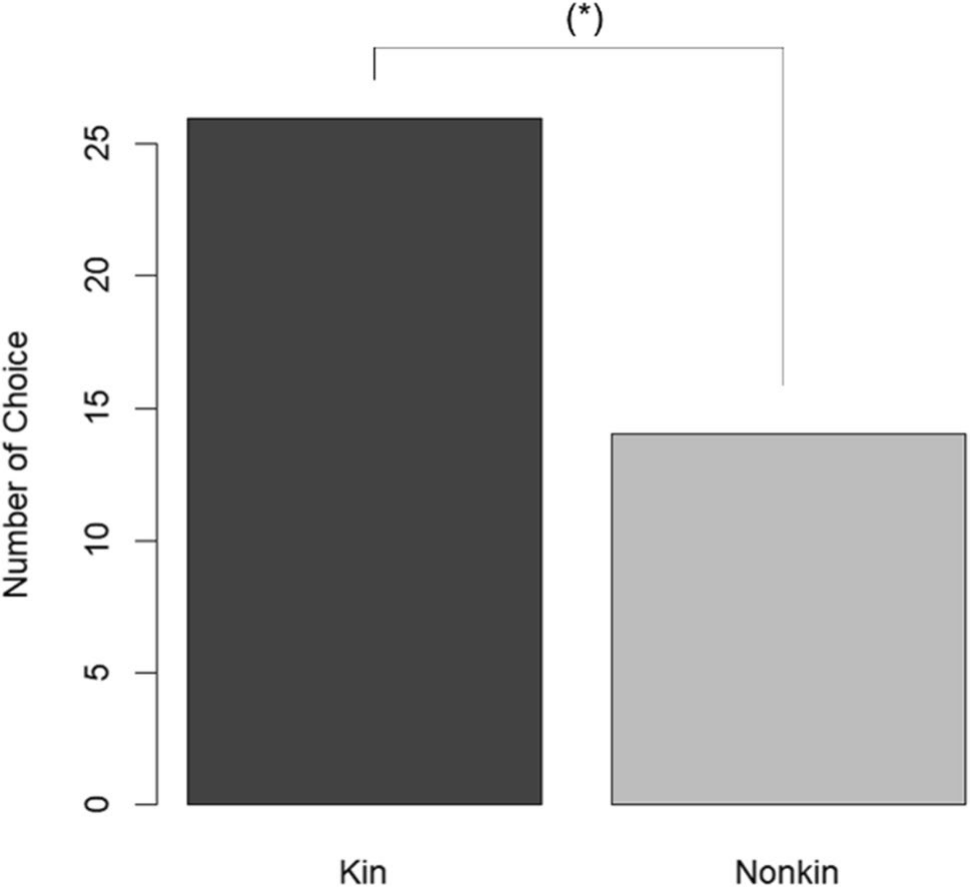
The choice of males during the experiment between related and unrelated females. Absolute values are shown. (*) indicates *p* < 0.1

Genetic relatedness tended to affect the mating pattern (GLMM, *z* = − 1.745, *p* = 0.081). Unfamiliar related females were chosen by 26 males, and unrelated females were chosen by 14 males (Fig. 3).

**Fig. 4:**
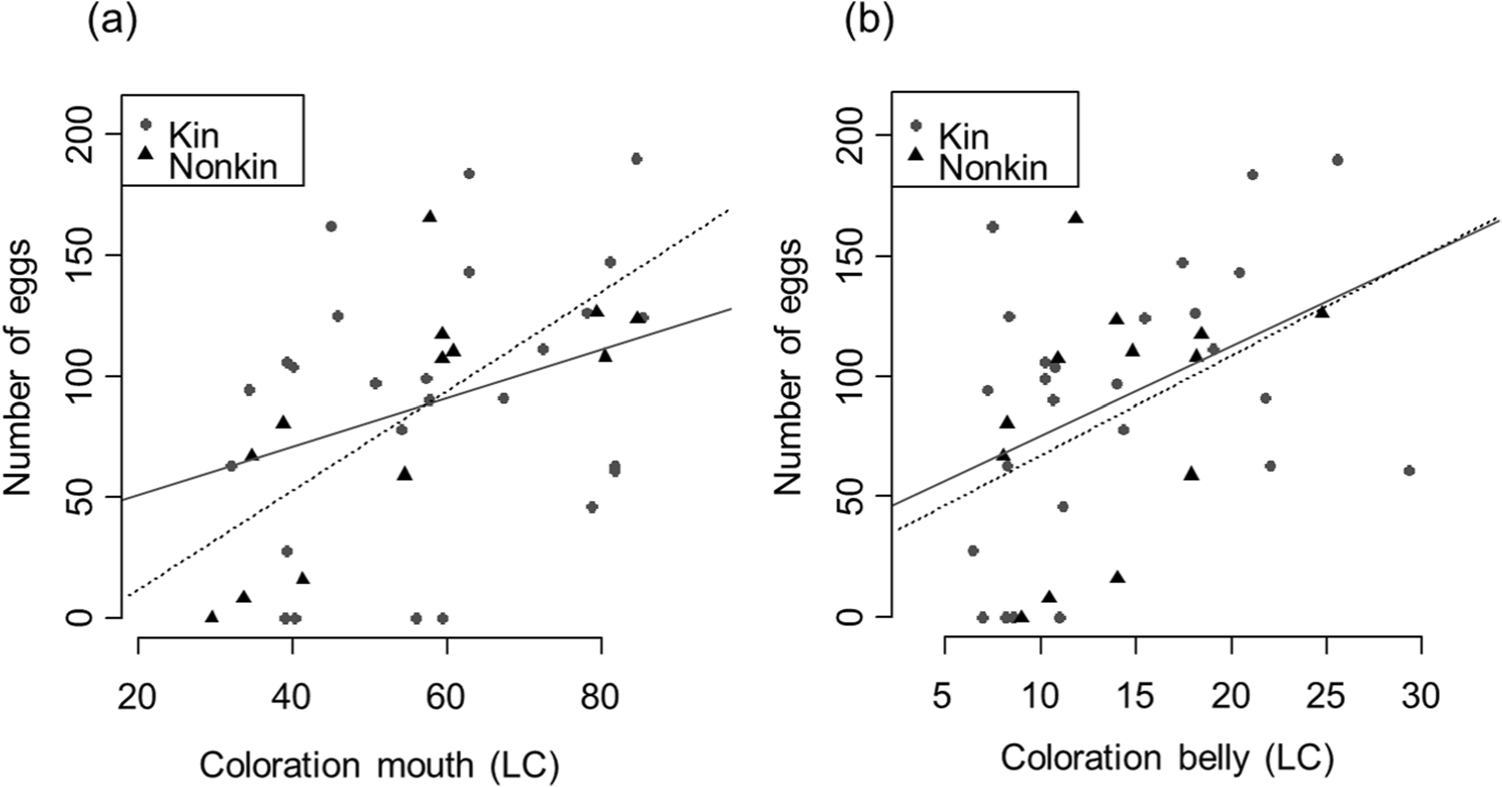
Relationship between **a** female mouth coloration (LC) and number of eggs and **b** female belly coloration (LC) and number of eggs are shown for related (shown with circles) and unrelated (shown with triangles) females. The lines show linear regression lines (least squares; solid line for kin, fine dashed for non-kin)

There was no significant difference between related and unrelated females in the days until a choice was made by the male (LMM, *t* = 0.994, *p* = 0.328). All breeding pairs spawned after pair formation, and there was no significant difference in the number of eggs between related and unrelated breeding pairs (LMM, *t* = − 1.054, *p* = 0.299). But there was a significant, positive correlation between the number of eggs and the females’ standard length (Table 1) and coloration (Table 1, Fig. 4). The number of eggs increased with the female’s body size.

**Table 1:** Linear mixed effects models calculated for the number of eggs, survival rate of clutches, and female-female aggression in relation to male-female relatedness, female size, female coloration (LC belly, LC mouth), female courtship and male choice

Dependent variable	Explanatory variable	***R*² (marg.)**	**Estimate**	***F***	***p***
*Number eggs*	Relatedness	0.174	− 12.147	0.545	0.465
	LC belly		3.316	6.479	**0.015**
*Number eggs*	Relatedness	0.193	− 8.229	0.253	0.618
	LC mouth		1.182	8.150	**0.007**
*Number eggs*	Relatedness	0.207	− 10.240	0.396	0.533
	SL		81.280	7.385	**0.009**
*Survival rate*	LC belly	0.017	0.001	0.024	0.877
	Relatedness		0.062	0.575	0.453
*Survival rate*	LC mouth	0.017	− 0.001	0.091	0.765
	Relatedness		0.057	0.575	0.453
*Survival rate*	SL	0.016	− 0.035	0.048	0.828
	Relatedness		0.059	0.575	0.452
*Survival rate*	Courtship	0.159	0.032	4.839	**0.040**
*Female aggression*	SL	0.152	0.665	0.560	0.461
	Relatedness		− 0.322	1.214	0.281
	Choice (yes/no)		0.685	2.697	0.112
*Female aggression*	Choice (yes/no)	0.277	0.416	0.701	0.410
	Relatedness		− 0.363	1.547	0.225
	LC mouth		0.022	6.408	**0.018**
*Female aggression*	Choice (yes/no)	0.452	0.299	0.442	0.515
	Relatedness		− 0.369	1.592	0.219
	LC belly		0.114	17.107	**< 0.001**

**Fig. 5:**
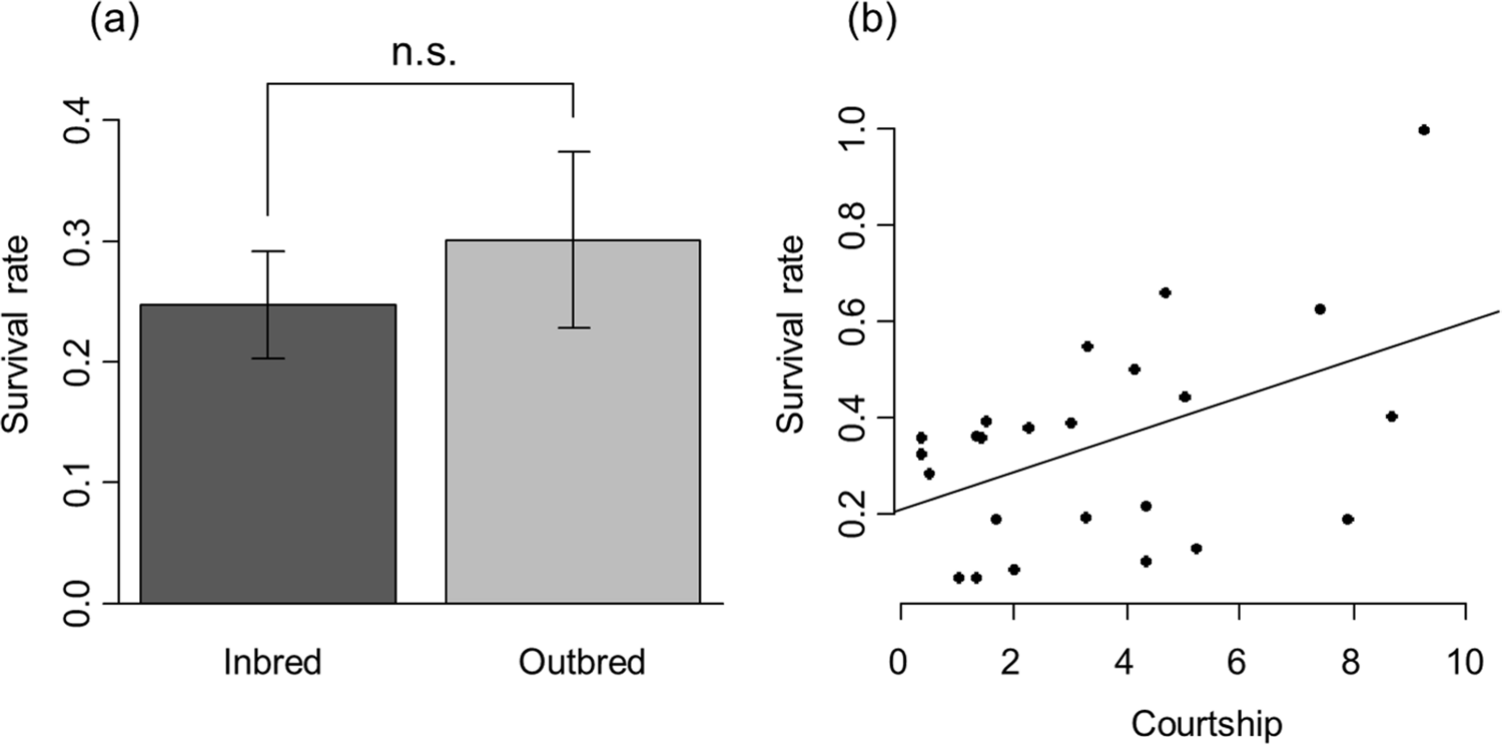
**a** Differences in the survival rate of offspring between inbred and outbred offspring. Mean values ± SE are shown. **b** Relationship between survival rate of offspring and the courtship behavior of females is shown. Line shows linear regression line (least squares)

**Fig. 6:**
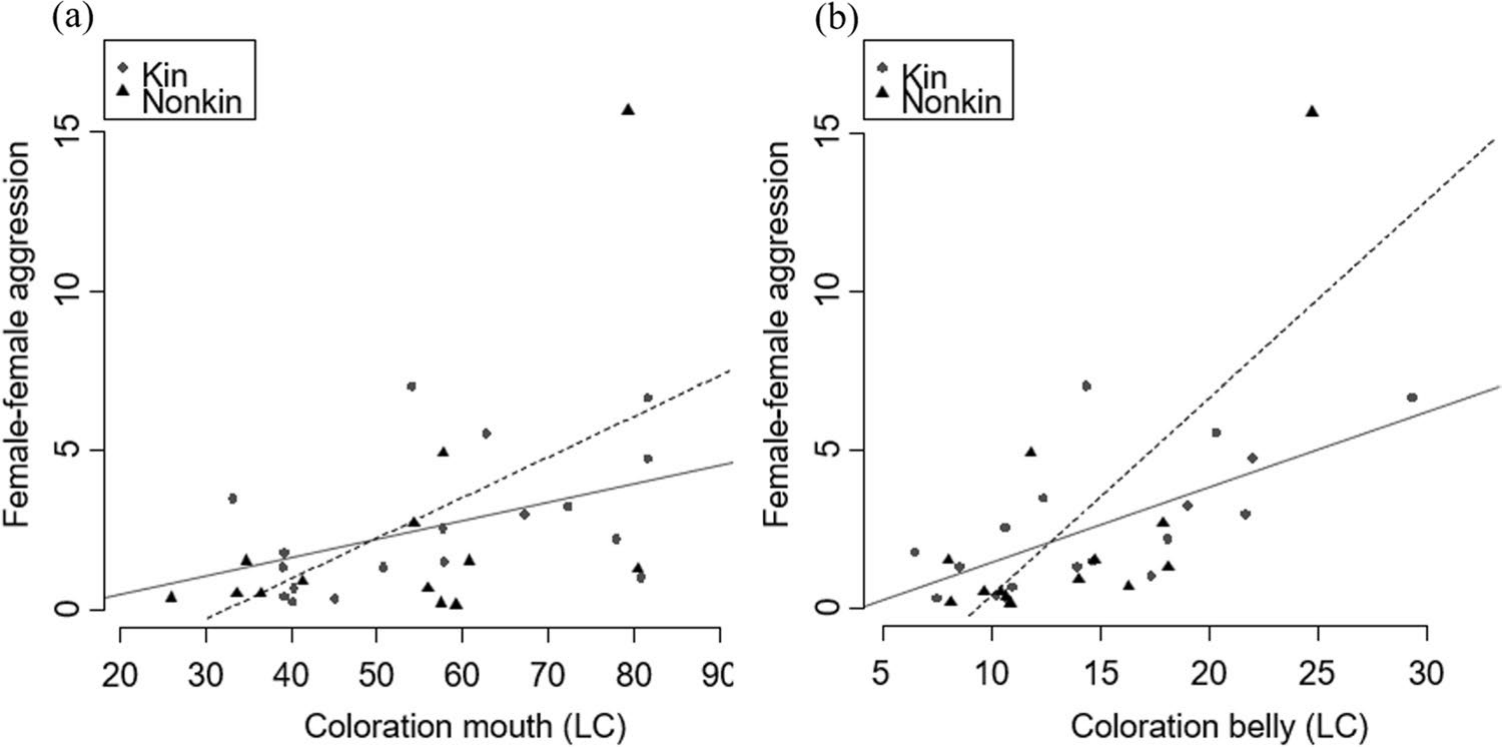
**a** Relationship of female aggressiveness and the female coloration of the mouth (LC), and **b** relationship between the female aggressiveness and the coloration of the belly (LC) for related (shown with circles) and unrelated (shown with triangles) females is shown. Lines show linear regression lines (least squares; solid lines show kin, fine dashed lines show non-kin)

There was no significant difference between inbred and outbred offspring survival rate (LMM, *t* = 0.744, *p* = 0.461; Fig. 5a). Neither female body coloration nor standard length correlated significantly with the offspring survival rate (Table 1). However, the survival rate of offspring was significantly positively influenced by the courtship behavior of females (Table 1; Fig. 5b). Female courtship behavior was not significantly different between related and unrelated females (LMM, *t* = − 0.529, *p* = 0.599). There was no significant difference in female-female aggression between related and unrelated females (Table 1), and there was furthermore no difference in male aggression directed towards related or unrelated females (Table 2). Female-female aggressiveness was significantly correlated with the intensity of the female’s coloration of the mouth and the belly (Table 1). The female-female aggressiveness and the intensity of coloration of the different body parts have a positive relationship (Fig. 6). Female size, coloration and courtship behavior did not significantly affect male choice; however, there was a statistical trend that more aggressive females were preferred by males (Table 3).

**Table 2:** Generalized mixed effects models calculated for aggression in relation to male-female relatedness and male choice

Dependent variable	Explanatory variables	*R*² (marg.)	estimate	*Z*	*p*
*Male aggression*	Relatedness	0.002	0.104	1.090	0.276
*Male aggression*	Choice (yes/no)	0.003	− 0.116	− 1.758	0.788

**Table 3:** Generalized mixed effects models calculated for the male choice in relation to female size, coloration (LC belly, LC mouth), courtship and aggression

Dependent variable	Explanatory variable	*R*² (marg.)	Estimate	*Z*	*p*
*Choice*	SL	0.002	0.232	0.261	0.794
*Choice*	LC belly	0.022	0.038	0.784	0.433
*Choice*	LC mouth	< 0.001	− 0.003	− 0.130	0.896
*Choice*	Courtship	0.784	− 0.375	− 1.769	0.168
	Female aggression		0.992	1.379	*0.077*

## Discussion

The aim of this study was to investigate the mating behavior with respect to genetic relatedness in *P. pulcher*. The results of this study showed no evidence for inbreeding avoidance but rather suggest inbreeding preference. Twenty-six out of 40 males chose the closely related female over the unrelated female during pair formation. Because mating with close relatives may lead to several fitness-related costs in fishes, such as reduced growth (Gallardo & Neira, 2005) or reduced offspring survival (Fessehaye et al., 2007), we aimed to identify differences between inbred and outbred offspring. In the F1, we found in one experiment a significant negative impact of inbreeding on juvenile survival. It is important to note that the offspring of that experiment resulted from “forced” matings with no choice options for the parents (brother-sister breeding pair or unrelated breeding pair) and that the broods were raised without parents from egg stage onwards (unpublished data). In the present experiment, there was no significant difference in the survival rate between inbred and outbred offspring. These findings are similar to those of a comparable trio experiment in the F1 with smaller sample size where we found no inbreeding avoidance (10 kin matings and 10 non-kin matings) and no significant inbreeding effect on juvenile survival (unpublished data). In both trio experiments (F1 and F2), males had a choice between 2 females (related or unrelated), and the resulting offspring was reared with their parents. This suggests that parental care buffers inbreeding depression as shown in ambrosia beetles (Pilakouta et al., 2015). In *P. pulcher*, both sexes intensely care for the brood, which includes fanning the eggs, removal of dead eggs, and cleaning the eggs. Also, the juveniles feed on the skin mucous of the parents in *P. taeniatus*, which may include immunologically relevant substances (Salinas et al., 2021). Parental care might be especially relevant for the survival of inbred individuals. Additionally, animals may be able to “identify” incompatible kin-mating partners to avoid producing poor quality offspring.

The number of eggs a female had spawned was significantly influenced by the standard length of females as well as the intensity of the body coloration of females. The number of eggs also increased with increasing body length and intensity of body coloration. Since female *P. pulcher* have been shown to use their bright coloration to compete for males and threat other females (Drennan, 2006), body size as well as coloration may signal dominance and individual quality (Baldauf et al., 2009). Also, in the closely related *P. taeniatus*, the intensity of coloration is known to function as an important signal during mating as well as during intra-sexual communication (Baldauf et al., 2009, 2010, 2013).

Furthermore, we aimed to analyze the influence of behavioral differences on mate choice behavior. In this study, there was no difference in the courtship behavior of related or unrelated females that was observed. Additionally, there was no difference in aggressiveness between the males towards chosen or not chosen females and no difference in the aggressiveness of males towards related or unrelated females. Nevertheless, the aggression between females was correlated with the mating decision. In fact, more aggressive females were more frequently selected as mates. This indicates that female-female competition plays an important role during mate choice decision. Because both parents invest relatively equally in raising their offspring, theory suggests that in monogamous, biparental cichlid fish, both males and females should be choosy (Barlow, 2000). Therefore, it is difficult to determine if the choosiness of one sex is decisive during the mate choice experiment.

We also showed a correlation between the intensity of the female’s body coloration and the female-female aggressiveness. A similar result was found in male *P. taeniatus* where aggression correlated positively with yellow intensity in dominant individuals (John et al., 2021). This again aligns with previous findings that the coloration in cichlid fish can signal dominance (Baldauf et al., 2009, 2010).

Survival rate of offspring was correlated with the intensity of the courtship behavior of females. Although this result would not remain significant after Bonferroni correction, similar results were found in other species. Hoikkala et al. (1998) showed in *Drosophila montana* that the frequency of male courtship displays correlated positively with the survival rate of the male’s progeny from the egg to adulthood which indicates an indirect benefit for the mating partner. Also, Knapp and Kovach (1991) found a positive correlation between the male’s courtship rate and the subsequent survival of eggs in the bicolor damselfish (*Stegastes partitus*). In that study, females with a more intensive courtship behavior were chosen more often, which indicates that intense courtship behavior may be an honest signal for quality. Future studies should analyze the influence of the female’s and male’s courtship behavior on the survival rate of offspring as well as the parental care.

In conclusion, this study showed no evidence for inbreeding avoidance or inbreeding depression in *P. pulcher*. The results of the present experiment rather suggest inbreeding preference as shown in *P. taeniatus*. The impact of parental care on buffering negative inbreeding effects will be examined in future studies. Furthermore, to get a comprehensive understanding of the consequences of inbreeding, further studies should also analyze the potential inbreeding effects on different traits in juveniles (e.g., growth, collective behavior, and cognitive abilities) and adults (e.g., fertility/fecundity, courtship, parental behavior, and ornamental traits). Since we found a positive correlation between female coloration and the number of eggs, the intensity of coloration may be an honest signal for female quality. Future studies should analyze the impact of male and female coloration on inter- and intra-sexual selection.

## Supplementary Information

Supplementary file1 (DOCX 13.9 KB)

Supplementary file1 (XLSX 53.3 KB)

## Data Availability

Data are provided as electronic supplementary material.

## References

[CR1] Andersson M, Simmons LW (2006) Sexual selection and mate choice. Trends Ecol Evol 21:296–302. 10.1016/j.tree.2006.03.015 10.1016/j.tree.2006.03.015 16769428

[CR2] Baldauf SA, Bakker TCM, Herder F, Kullmann H, Thünken T (2010) Male mate choice scales female ornament allometry in a cichlid fish. BMC Evol Biol 10:1–9. 10.1186/1471-2148-10-301 10.1186/1471-2148-10-301 PMC2958921 20932273

[CR3] Baldauf SA, Engqvist L, Ottenheym T, Bakker TCM, Thünken T (2013) Sex-specific conditional mating preferences in a cichlid fish: implications for sexual conflict. Behav Ecol Sociobiol 67:1179–1186. 10.1007/s00265-013-1543-4

[CR4] Baldauf SA, Kullmann H, Thünken T, Winter S, Bakker TCM (2009) Computer animation as a tool to study preferences in the cichlid *Pelvicachromis taeniatus*. J Fish Biol 75:738–746. 10.1111/j.1095-8649.2009.02347.x 10.1111/j.1095-8649.2009.02347.x 20738572

[CR5] Ballou J (1983) Calculating inbreeding coeffients from pedigrees. In: Schonewald-Cox CM, Chambers SM, MacBryde B, Thomas L (eds) Genetics and conservation. Benjamin/Cummings, Menlo Park, pp 509–520

[CR6] Barlow GW (2000) The cichlid fishes: nature’s grand experiment in evolution. Perseus, Cambridge

[CR7] Bateman AJ (1948) Intra-sexual selection in *Drosophila*. Heredity 2:349–368 10.1038/hdy.1948.21 18103134

[CR8] Bates D, Mächler M, Bolker BM, Walker SC (2015) Fitting linear mixed-effects models using lme4. J Stat Softw 67:1–48. 10.18637/jss.v067.i01

[CR9] Bolger T, Connolly PL (1989) The selection of suitable indices for the measurement and analysis of fish condition. J Fish Biol 34:171–182. 10.1111/j.1095-8649.1989.tb03300.x

[CR10] Brown JL, Bush M, Packer C, Pusey AE, Monfort SL, O’Brien SJ, Janssen DL, Wildt DE (1993) Hormonal characteristics of free-ranging female lions (*Panthera leo*) of the Serengeti Plains and Ngorongoro crater. J Reprod Fertil 97:107–114. 10.1530/jrf.0.0970107 10.1530/jrf.0.0970107 8385220

[CR11] Charlesworth D, Charlesworth B (1987). Inbreeding depression and its evolutionary consequences. Annu Rev Ecol Syst.

[CR12] Chen Y, Hao P (2004). Optimal transform in perceptually uniform color space and its application in image retrieval. International Conference on Signal Processing Proceedings, ICSP.

[CR13] Clutton-Brock TH, Vincent ACJ (1991) Sexual selection and the potential reproductive rates of males and females. Nature 351:58–60. 10.1038/351058a0 10.1038/351058a0 2027382

[CR14] Craig JK, Foote CJ (2001) Countergradient variation and secondary sexual color: phenotypic convergence promotes genetic divergence in carotenoid use between sympatric anadromous and nonanadromous morphs of sockeye salmon (*Oncorhynchus nerka*). Evolution 55:380–391. 10.1111/j.0014-3820.2001.tb01301.x 10.1111/j.0014-3820.2001.tb01301.x 11308094

[CR15] Daniel MJ, Rodd FH (2016) Female guppies can recognize kin but only avoid incest when previously mated. Behav Ecol 27:55–61. 10.1093/beheco/arv122

[CR16] Darwin C (1871). The descent of man, and selection in relation to sex.

[CR17] Drennan LM (2006) Female competition and display in kribensis (*Pelvicachromis pulcher*), a West African cichlid. Master thesis, California State University

[CR18] Duthie AB, Lee AM, Reid JM (2016) Inbreeding parents should invest more resources in fewer offspring. Proc R Soc B 283(1843):20161845 10.1098/rspb.2016.1845 PMC5136589 27881747

[CR19] Fessehaye Y, Komen H, Rezk MA, van Arendonk JAM, Bovenhuis H (2007). Effects of inbreeding on survival, body weight and fluctuating asymmetry (FA) in Nile tilapia, *Oreochromis niloticus*. Aquaculture.

[CR20] Gallardo JA, Neira R (2005) Environmental dependence of inbreeding depression in cultured Coho salmon (*Oncorhynchus kisutch*): aggressiveness, dominance and intraspecific competition. Heredity 95:449–456. 10.1038/sj.hdy.6800741 10.1038/sj.hdy.6800741 16189545

[CR21] Gow EA, Arcese P, Dagenais D, Sardell RJ, Wilson S, Reid JM (2019) Testing predictions of inclusive fitness theory in inbreeding relatives with biparental care. Proc R Soc B 286:20191933. 10.1098/rspb.2019.1933 10.1098/rspb.2019.1933 PMC6939262 31795864

[CR22] Hamilton WD (1964). The genetical evolution of social behaviour. J Theor Biol.

[CR23] Hedrick PW (1994). Purging inbreeding depression and the probability of extinction: full-sib mating. Heredity.

[CR24] Herdman EJE, Kelly CD, Godin J-GJ (2004) Male mate choice in the guppy (*Poecilia reticulata*): do males prefer larger females as mates? Ethology 110:97–111. 10.1111/j.1439-0310.2003.00960.x

[CR25] Hoikkala A, Aspi J, Suvanto L (1998) Male courtship song frequency as an indicator of male mating success in *Drosophila montana*. J Insect Behav 12:599–609. 10.1023/A:1020971518710 10.1098/rspb.1998.0323 PMC1688912 9569668

[CR26] John L, Rick IP, Vitt S, Thünken T (2021) Body coloration as a dynamic signal during intrasexual communication in a cichlid fish. BMC Zool 6:1–13. 10.1186/s40850-021-00075-9 10.1186/s40850-021-00075-9 PMC10127425 37170176

[CR27] Knapp RA, Kovach JT (1991) Courtship as an honest indicator of male parental quality in the bicolor damselfish, *Stegastes partitus*. Behav Ecol 2:295–300. 10.1093/beheco/2.4.295

[CR28] Kokko H, Johnstone RA (2002) Why is mutual mate choice not the norm? Operational sex ratios, sex roles and the evolution of sexually dimorphic and monomorphic signalling. Philos Trans R Soc B 357:319–330. 10.1098/rstb.2001.0926 10.1098/rstb.2001.0926 PMC1692955 11958700

[CR29] Kokko H, Jennions MD, Brooks R (2006). Unifying and testing models of sexual selection. Annu Rev Ecol Evol Syst.

[CR30] Kokko H, Ots I (2006) When not to avoid inbreeding. Evolution 60:467–475. 10.1111/j.0014-3820.2006.tb01128.x 16637492

[CR31] Kuznetsova A, Brockhoff PB, Christensen RHB (2017) lmerTest package: tests in linear mixed effects models. J Stat Softw 82:1–26. 10.18637/jss.v082.i13

[CR32] Langen K, Schwarzer J, Kullmann H, Bakker TCM, Thünken T (2011) Microsatellite support for active inbreeding in a cichlid fish. PLoS ONE 6:e24689. 10.1371/journal.pone.0024689 10.1371/journal.pone.0024689 PMC3184091 21980351

[CR33] Lüdecke D, Ben-Shachar MS, Patil I, Makowski D (2021) Performance: an R package for assessment, comparison and testing of statistical models. J Open Source Softw 6:3139. 10.21105/joss.03139

[CR34] Martin E, Taborsky M (1997) Alternative male mating tactics in a cichlid, *Pelvicachromis pulcher*: a comparison of reproductive effort and success. Behav Ecol Sociobiol 41:311–319. 10.1007/s002650050391

[CR35] Meuthen D, Baldauf SA, Bakker TCM, Thünken T (2018) Neglected patterns of variation in phenotypic plasticity: age- and sex-specific antipredator plasticity in a cichlid fish. Am Nat 191:475–490. 10.1086/696264 10.1086/696264 29570404

[CR36] Nichols HJ (2017). The causes and consequences of inbreeding avoidance and tolerance in cooperatively breeding vertebrates. J Zool.

[CR37] Parker GA (1983) Mate quality and mating decisions. In: Bateson P (ed) Mate Choice, Cambridge University Press, Cambridge, pp 141–166

[CR38] Pike VL, Cornwallis CK, Griffin AS (2021) Why don’t all animals avoid inbreeding? Proc R Soc B 288:20211045. 10.1098/rspb.2021.1045 10.1098/rspb.2021.1045 PMC8334842 34344184

[CR39] Pilakouta N, Jamieson S, Moorad JA, Smiseth PT (2015) Parental care buffers against inbreeding depression in burying beetles. Proc Natl Acad Sci USA 112:8031–8035. 10.1073/pnas.1500658112 10.1073/pnas.1500658112 PMC4491787 26080412

[CR40] Pusey A, Wolf M (1996) Inbreeding avoidance in animals. Trends Ecol Evol 11:201–206. 10.1016/0169-5347(96)10028-8 10.1016/0169-5347(96)10028-8 21237809

[CR41] R Core Team (2021) R: a language and environment for statistical computing. Vienna, Austria. Retrieved from https://www.R-project.org/

[CR42] Radwan J (2003). Inbreeding depression in fecundity and inbred line extinction in the bulb mite, *Rhizoglyphus robini*. Heredity.

[CR43] Robertson AR (1977) The CIE 1976 color-difference formulae. Color Res Appl 2:7–11. 10.1002/j.1520-6378.1977.tb00104.x

[CR44] Salinas I, Fernández-Montero Á, Ding Y, Sunyer JO (2021). Mucosal immunoglobulins of teleost fish: a decade of advances. Dev Comp Immunol.

[CR45] Sargent RC, Gross MR, Van Den Berghe EP (1986) Male mate choice in fishes. Animal Behaviour 34:545–550. 10.1016/S0003-3472(86)80123-3

[CR46] Scherer U (2019). Does sexual selection favour consistent behavioural differences in bi-parental cichlids?.

[CR47] Scherer U, Schuett W (2018). No male mate choice for female boldness in a bi-parental West African cichlid, the rainbow krib (*Pelvicachromis pulcher*). PeerJ.

[CR48] Schlupp I (2021) Male choice, female competition, and female ornaments in sexual selection. Oxford University Press, Oxford. 10.1093/oso/9780198818946.001.0001

[CR49] Schons RF, Vitt S, Thünken T (2022) Resource heterogeneity but not inbreeding affects growth and grouping behaviour in socially foraging juvenile cichlid fish. Funct Ecol 36:550–560. 10.1111/1365-2435.13960

[CR50] Su GS, Liljedahl L-E, Gall GAE (1996) Effects of inbreeding on growth and reproductive traits in rainbow trout (*Oncorhynchus mykiss*). Aquaculture 142:139–148. 10.1016/0044-8486(96)01255-0

[CR51] Thünken T, Bakker TCM, Baldauf SA, Kullmann H (2007). Active inbreeding in a cichlid fish and its adaptive significance. Curr Biol.

[CR52] Thünken T, Meuthen D, Bakker TCM, Kullmann H (2010) Parental investment in relation to offspring quality in the biparental cichlid fish *Pelvicachromis taeniatus*. Anim Behav 80:69–74. 10.1016/j.anbehav.2010.04.001

[CR53] Trivers RL (1972) Parental investment and sexual selection. In: Campbell B (ed) Sexual selection and the descent of man, Aldine, Chicago, pp 136–179. 10.4324/9781315129266-7

[CR54] Vitt S, Bakowski CE, Thünken T (2022) Sex-specific effects of inbreeding on body colouration and physiological colour change in the cichlid fish *Pelvicachromis taeniatus*. BMC Ecol Evol 22:1–11. 10.1186/s12862-022-02074-x 10.1186/s12862-022-02074-x PMC9623988 36316663

[CR55] Waldmann B, McKinnon JS (1993) Inbreeding and outbreeding in fishes, amphibians, and reptiles. In: Thornhill NW (ed) The natural history of inbreeding and outbreeding: theoretical and empirical perspectives, University of Chicago Press, Chicago, pp 250–282

[CR56] Willoughby JR, Waser PM, Brüniche-Olsen A, Christie MR (2019) Inbreeding load and inbreeding depression estimated from lifetime reproductive success in a small, dispersal-limited population. Heredity 123:192–201. 10.1038/s41437-019-0197-z 10.1038/s41437-019-0197-z PMC6781135 30809076

