# Supplementary material for: Impact of genetic relatedness on reproductive behavior in Pelvicachromis pulcher, a biparental cichlid fish with mutual mate choice and ornamentation
Source: Naturwissenschaften. 2023 May 4;110(3):17. doi: 10.1007/s00114-023-01842-z (PMC10160185; doi:10.1007/s00114-023-01842-z)
Supplement: Supplementary file 1 — Supplementary file1 (DOCX 13.9 KB) [file 114_2023_1842_MOESM1_ESM.docx]

| Model | GLMM/LMM | Dependent variable | Explanatory variables | Random effects |
| --- | --- | --- | --- | --- |
| M1 | LMM | Number of Eggs | Relatedness + LC belly | (1\|Tank) + (1\|Family F) + (1\|Family M/ID M) + (1\|Feeding regime) |
| M2 | LMM | Number of Eggs | Relatedness + LC mouth | (1\|Tank) + (1\|Family F) + (1\|Family M/ID M) + (1\|Feeding regime) |
| M3 | LMM | Number of Eggs | Relatedness + SL | (1\|Tank) + (1\|Family F) + (1\|Family M/ID M) + (1\|Feeding regime) |
| M4 | LMM | Survival rate | Relatedness + LC belly | (1\|Tank) + (1\|Family F) + (1\|Family M/ID M) + (1\|Feeding regime) |
| M5 | LMM | Survival rate | Relatedness + LC mouth | (1\|Tank) + (1\|Family F) + (1\|Family M/ID M) + (1\|Feeding regime) |
| M6 | LMM | Survival rate | Relatedness + SL | (1\|Tank) + (1\|Family F) + (1\|Family M/ID M) + (1\|Feeding regime) |
| M7 | LMM | Survival rate | Courtship | (1\|Family F) |
| M8 | LMM | Female aggression | Relatedness + Choice + SL | (1\|Tank) + (1\|Family F) + (1\|Family M/ID M) + (1\|Feeding regime) |
| M9 | LMM | Female aggression | Relatedness + Choice + LC mouth | (1\|Tank) + (1\|Family F) + (1\|Family M/ID M) + (1\|Feeding regime) |
| M10 | LMM | Female aggression | Relatedness + Choice + LC belly | (1\|Tank) + (1\|Family F) + (1\|Family M/ID M) + (1\|Feeding regime) |
| M11 | GLMM | Choice | 1 | (1\|Tank) + (1\|Family M/ID M) + (1\|Feeding regime) |
| M12 | GLMM | Choice | Diff SL | (1\|Trial) + (1\|Family F) + (1\|Family M) |
| M13 | GLMM | Choice | Diff LC belly | (1\|Trial) + (1\|Family F) + (1\|Family M) |
| M14 | GLMM | Choice | Diff LC mouth | (1\|Trial) + (1\|Family F) + (1\|Family M) |
| M15 | GLMM | Choice | Diff Aggression F + Diff Courtship F | (1\|Trial) + (1\|Family F) + (1\|Family M) |
| M16 | GLMM | Male aggression | Relatedness | (1\|Trial) + (1\|Family F) + (1\|Family M/ID M) + (1\|Feeding regime) |
| M17 | GLMM | Male aggression | Choice | (1\|Trial) + (1\|Family F) + (1\|Family M/ ID M) + (1\|Feeding regime) |

Gussone et al. Supplementary 1: All models used in this study. F= Female, M= Male
